# Frontal bone hyperostotic mass associated with fibrous dysplasia in a male patient with systemic lupus erythematosus (SLE)

**DOI:** 10.1016/j.ijscr.2021.106564

**Published:** 2021-11-18

**Authors:** Giorgio Giampaoli, Salvatore Chirumbolo, Paola Di Ceglie, Dario Bertossi, Riccardo Nocini

**Affiliations:** a Department of Surgery, Dentistry, Paediatrics and Gynaecology, Unit of Maxillo-Facial Surgery, University of Verona, Verona, Italy; b Department of Neurosciences, Biomedicine and Movement Sciences, University of Verona, Verona, Italy; c Department of Surgery, Dentistry, Paediatrics and Gynaecology, Unit of Otorhinolaryngology, University of Verona, Verona, Italy

**Keywords:** Hyperostosis, Piezoelectric surgery, Hemangioma, Head and neck

## Abstract

We present a rare clinical report of a 45-year-old man affected by Systemic Lupus Erythematosus (SLE) with a unilateral mass of the left frontal bone, diagnosed as a possible recurrence of fibrous dysplasia. This patient was evaluated with computed tomography (CT scan) and was treated with resection of the mass and reconstruction with splitting a calvarian bone graft. The pathological evidence was suggestive for a bone cavernous haemangioma.

No previously described cases of bone dysplasia associated with this systemic syndrome were reported so far. Patient's disease was under stringent control at the time of hospitalization, and the outcome has been successful, even though a mild increase of inflammatory indexes was reported after surgery. This laboratory evidence was transient and not related to further clinical complications.

## Highlights

• Piezoelectric surgery allowed us to surgically treat a hemangioma.
• Hyperostosis found in the case report was due to hemangioma.
• Hyperostotic cases can be treated with piezosurgery.
• Case report with SLE

## 1 Introduction

Hyperostosis is a wide terminology used to indicate an abnormality in bone growth, i.e. in the increasing of ossification process in the skeleton, which should not be applied to such adaptive changes in size and mass of bones associated with an enhanced mechanical work [1]. Briefly speaking, hyperostosis is commonly defined as an excessive bone growth enabled in altering the normal subject's anatomy. A focal growth is usually found in disorders such as fibrous dysplasia (FD), meningiomas, ossifying fibroma, or hyperostosis frontalis interna (HFI) [2], [3]. A case of hyperostosis in systemic lupus erythematosus (SLE) has been also reported quite recently [4].

Fibrous dysplasia (FD) of bone is a congenital condition, which can affect either a single bone (monostotic form) or multiple bones (polyostotic form), with frequently unilateral distribution. The underlying molecular abnormality concerns somatic mutation of the *GNAS* gene, resulting in osteoblast differentiation deficit, fibrous medullary proliferation and osteoclast hyperactivity partly due to over-expression of IL-6 within transformed cells [5], [6]. Moreover, diffuse hyperostosis (multiple distribution of bone excess) has been reported in several disorders like Paget's disease, acromegaly, osteopetrosis and hyperparathyroidism [7], [8].

Cranial and skull hemangionmas, often described as primary intraosseous cavernous hemangiomas (PICHs) were recently described, though occurring in a lesser extent in skull than in spine [9]. Toynbee was the first physician in having described PICHs in 1845 as vascular tumors growing at the boundaries of the skull bones [9]. Some case report of cranial hemangioma was described in the past, although very rarely in SLE patients [10].

Cranial-facial involvement in FD is found in 50% of polyostotic and 27% of monostotic forms [11], [12]. Actually, FD is considered a benign but progressive bone lesion, arisen as a replacement of normal medullar bone with extensive fibrous tissue. It usually tends to be monostotic, with a single area of bone involvement, and often presents itself as a painless, slow growing mass with swelling of skull bones [11], [12]. The majority of FD cases are not reported because they usually show clear symptomatology upon lesion growing rapidly or when other accessory symptoms occur (e.g. events causing nerve compression). In this sense, FD can be considered the most common form of hyperostosis, for which biased interpretations may occur and often surgical intervention is not suggested or recommended.

Typical symptoms of frontal bone fibrous dysplasia are mild headaches, neurological symptoms or vision changes in worst manifestations were reported [12], [13]. Furthermore, *GNAS* gene encodes the α-subunit of the stimulatory heterotrimeric G protein (Gsα) and has been associated with heterotopic ossification [14], [15]. To date, no sound evidence, at the best of our knowledge, was reported associating *GNAS* mutations (and FD) with SLE, despite some anecdotal cases [4].

## 2 Case report

**Fig. 1:**
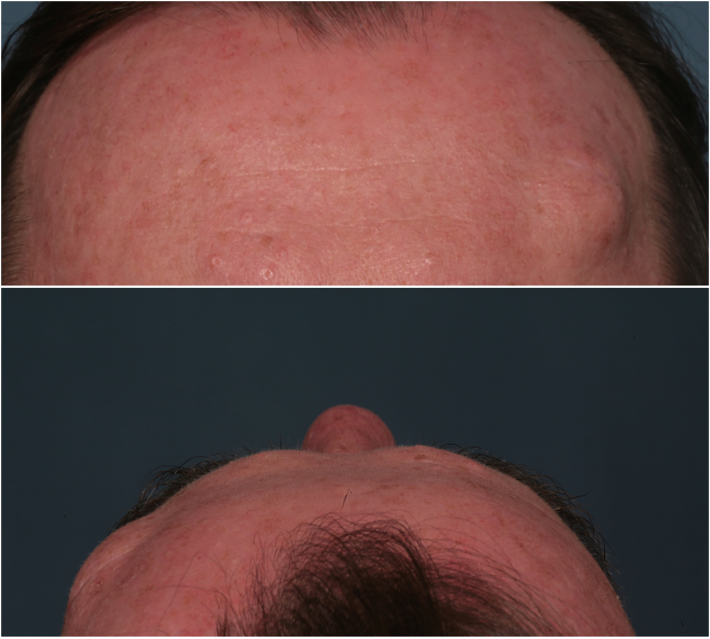
Image of case report with hyperostoti mass. The hyperostotic bulge can be observed on the right (in the photo), left (for the patient) both in the frontal (top) and upper transversal (down) images.

A 45 years old man, affected by SLE, came to our attention for a thick painless frontal mass with a 30 × 20 mm extension located on his left forehead (Fig. 1). The patient signed an informed consent before accessing our services and was actively admitted in the research study (Supplementary Material S1). He referred a slowly but progressive growing during the latest year. In anamnesis he referred no previous illnesses, moreover SLE was under control. He denied allergies and he was not up-taking any therapy. Moreover, he was not under immuno-modulatory treatment. He suffered from a similar mass growth previously, located nearby the current area in his frontal bone and which was surgically excised two years before in local anesthesia, because diagnosed as FD. He reported neither neurological symptom, nor even recurrent headaches.

The patient had a CT-scan suggested to assess a sclerotic form of FD, which resulted in a ground-glass appearance of a homogenous matrix expansion within the skeleton, affecting both bone plaques, particularly the external cortex (Report in Supplementary Materials S2). Imaging was unable to assess any presumptive diagnosis; a pathology investigation was attempted to exclude the existence of a malignant neoplasm underneath. Therefore, the patient was evaluated by fine needle aspiration cytology (FNAC) and optical immuno-staining, which excluded the presence of CD138^+^ cells for multiple myeloma diagnosis (Supplementary Material S3). Furthermore, the histological analysis described a diffuse sclerosis and complete resorption of bone, characterized by intra-trabecular fibrosis. As the bone did not appear uniformly mineralized, findings were suggestive for FD.

Surgical excision of FD-related bone disorders, is considered the elective and recommended treatment and must be provided with at least 10 mm of healthy tissue margins [11]. Pre-operative laboratory assays reported no inflammatory status (CRP and ESR, eGRF and cell blood with the normality range).

**Fig. 2:**
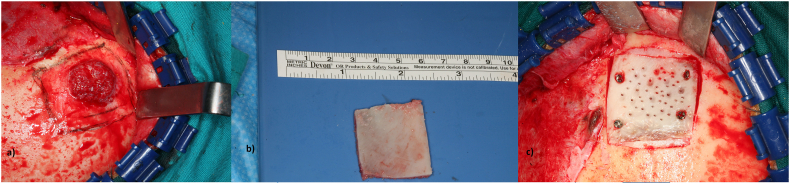
Evidence of hemangioma during surgery. From left to right: 1) Bone haemangioma with coronal access, 2) Calvarian graft, 3) surgical reconstruction.

A complete resection was performed, a 40 × 50 mm portion of frontal bone was excised, which included both the identified bone mass and 15 mm rim of normal tissue. Piezoelectric surgery was used for osteotomies and the defect was repaired with an autologous bone graft from calvaria, fixed with cortical screws. Moreover, the excision was performed with coronal approach in order to preserve aesthetic features hiding the surgical scar under the scalp. (Fig. 2).

Post-op was normal, no side was detected, in 3 days the patient was discharged with a small increase in the major inflammatory serum markers (ESR from 8 mm/h to 32 mm/h, CRP from 4 mg/L to 9 mg/L, WBC 18.780/μl, with marked neutrophilia (17.310/μl), related to post-op course.

Upon anatomical-pathological investigation, the surgically excised mass has been recognized as a bone cavernous haemangioma.

**Fig. 3:**
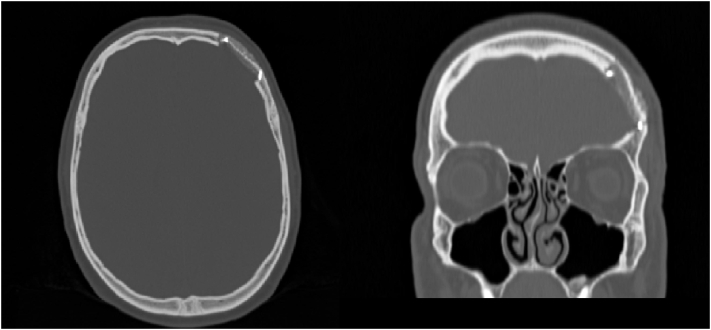
Post-operative (post-op) computing tomography (CT) scan. Left: Transversal and (right) frontal plane. Note the surgical area lacking of the hyperostotic mass.

**Fig. 4:**
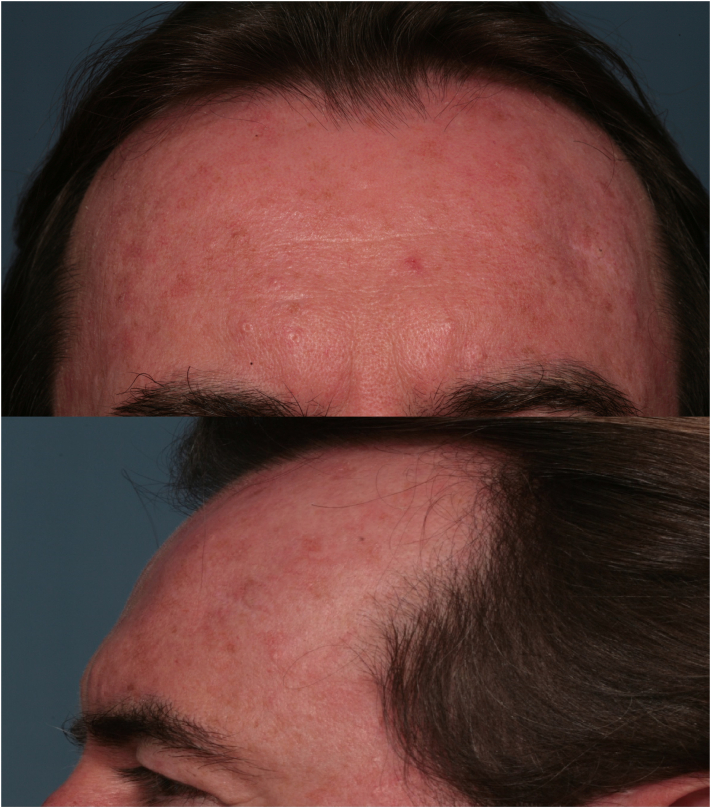
Post-op photos of the case report: Top: Frontal image, down: lateral image. The hyperostotic mass is disappeared.

Patient's follow up was set at 6 and 12 months, outcome reported no recurrence, symptoms and further adverse signs. A further CT scan was not additionally asked in order to avoid an excessive ionizing radiation exposure, since cavernous haemangiomas are recognized as non-malignant tumors. (Fig. 3, Fig. 4).

## 3 Discussion

FD diagnosis represents a common source of bias, by usually observing FD-related hyperostosis. Actually, hyperostotic masses can be related to a wide variety of disorders, each of those presenting a typical clinical upset and specific pathological evidence. FD is histologically characterized by overgrowth of the fibrous stroma surrounding disorganized trabeculae, thinning of the bone cortex and dysplastic bony spicules without osteoblasts, then resulting in woven bone that appears unable to transforming into lamellar bone. Differential diagnosis should be performed on the basis of bone increase patterns. For example, Paget's disease may be described into 3 phases, which usually exist separately or can be found in the same bone simultaneously, i.e. osteoclastic osteolysis, osteoblastic-osteolytic intermediate phase and sclerotic phase performed mainly by osteoblasts that show increased activity. Therefore, Paget disease is typically related to a histological mosaic pattern due to the disorganization of the lamellar bone structure. On the other hand, acromegaly is an endocrine disorder due to an impairment in the GH biology. Clinical manifestations of this disease are specific and highly suggestive for a definitive diagnosis, including frontal bossing, macroglossia, exaggerated growth of hands and feet, hypertrophy of the mandible and thickening of the cortical bone. Furthermore, osteofibrous dysplasia and ossifying fibroma are respectively identified with global thickening of the tibia and of the jaw. Contrarily to FD, osteofibrous dysplasia is a mixture of woven and lamellar bone, and is also characterized by a zonal architecture with central immature trabeculae of woven bone gradually progressing into an outer zone of mature and lamellar bone. Finally, osteopetrosis type 1 shows a progressive thickening of the entire skull, with dense sclerosis of both tables and widening of the diploic space. Other hyperostotic masses with local extension can be related to affections as endosteal osteoma, osteosarcoma, meningiomas, or metastatic tumors [16].

Intra-osseous hemangiomas are benign primary neoplasms arising from the intrinsic vasculature of the bone and represent 0,2% of bone tumors [17]. They can be congenital or traumatic. Differently from hemangioma located in the maxilla or in the jaw, they rarely show artery-venous anastomoses. Most common skull hemangioma are of cavernous types, these are usually not related to aberrant vessels and can be excised with low intra-operative risks [17]. Recent literature has reported cavernous hemangiomas in SLE, though involving liver [18], [19].

These kinds of tumors, are extremely rare and often their localization is hard to be detected by CT scan because mesenchymal vessel tissue is similar to matrix deposits typical of FD. Furthermore, cavernous haemangioma are characterized by low-flow vessels abnormalities, then fine needle aspiration cytology (FNAC) cannot detect any specific anomalies. [20], [21].

Regarding surgical treatment, usually, osteotomies are provided by rotating instruments, which needs caution, as they produce heat due to friction forces. Piezoelectric bone surgery is a technique of osteotomy and osteoplasty, which requires the use of micro-vibrations of ultrasonic frequency scalpels. The principle of piezosurgery is ultra sound (US) transduction [22], obtained by piezoelectric ceramic contraction and expansion. The vibrations are amplified over the bone and results in the presence of irrigation with physiological solution, in the cavitation phenomenon, with a mechanical cutting effect exclusively on mineralized tissues, preserving soft tissues (such as periostium) from injuries, and thus enhancing a faster recovery [23]. Periostium provides blood supply which is fundamental for bone integration of the autologous graft and to avoid peri-operative complications, such as infections, dehiscence and delayed wound healing [24].

Defect replacement can be performed using an autologous bone graft or alloplastic material, for example titanium mesh. Although alloplastic materials and bone substitutes have been used for cranial reconstruction, the best option is autologous bone. In contrast to synthetic materials, autologous graft grants a faster osteo-integration, due to their osteogenic, osteoinductive and osteconductive properties. Therefore, alloplastic materials should be considered a good alternative only for smaller bone defects reconstruction. Moreover, using calvarian bone grafts reported lower incidence of donor site comorbidity, comparing to hip or ribs grafts, and better integration with skull bones [25].

It has been reported that SLE patients have increased risk of postoperative complications after major surgery, those mostly results in an increased rate of bleeding and infections, especially during hospitalization. Such studies concern major orthopedic surgery, because these patients are often affected by arthritis and they often undergo hips or knee arthroplasty [26], [27]. Post-operative course in maxillofacial surgery has not been investigated yet.

There are two major factors related to early and late complications, such as delayed healing: a basal inflammatory onset, typical for these patients, and prolonged therapies with steroids and/or immuno-regulators. Patients affected by SLE are often exposed to pharmacological side effects, which are more frequent with increasing therapy dosages. Thus, we can assume that complications are strictly related to the level of activity of their disease and the presence of comorbidities, for example arthritis, nephritis, ulcerative lesions or uveitis. Interestingly, nephritis has been recently associated with FD [28]. Moreover, postoperative course might trigger SLE in these kinds of patients because of the increasing secretion of inflammatory cytokines. [27].

Finally, SCARE guidelines were followed to report this case report [28], [29].

## 4 Conclusion

This is the first reported case of calvarian hemangioma in a LES patient, described as a hyperostotic mass.. Hyperostotic mass of the skull should be always investigated when it becomes symptomatic or clinically evident. FD is a benign condition that can affect craniofacial bones, and is not always needs to be surgically treated. Calvarian hemangioma has a benign course after excision. These lesions tend to grow, engaging cranial nerves especially when located in the skull base, then surgical treatment is strictly recommended. Their treatment consists in total excision including a rim of normal bone, and repair of the bone defect with autologous bone grafts or a titanius mesh, depending on the defect extension.

Piezosurgery should be always considered as first choice to perform osteotomies in reconstructive surgery: preserving periostium blood supply assures faster recovery and lower incidence of post-operative complications, avoiding bone and soft tissue secondary healing and re-modelling of anatomical surfaces. Thus, it grants also a better aesthetic outcome.

Even though the excision of bone mass would require a CT imaging follow up, clinical evaluation after 6 months and every year afterwards, in absence of symptoms, is enough to exclude a relapse of a rare benign neoplasm such as cavernous hemangiomas of the skull. Morphological changes can be detected by the surgeon during clinical investigation, avoiding periodical high dose x-ray expositions. Obviously, CT imaging should be always considered when a bone neoplasm in the same area is detected, as well as if patient complains headaches, vision changes or neurological symptoms.

Patients affected by SLE undergo more often post-operative sides, due both to over-reactive immune responses and consequences of prolonged steroid and/or immuno-regulative therapies. Therefore, it may be necessary to define the SLE grading during surgery planning to assure fast recovery and to avoid occurrence of complications.

## Sources of funding

None.

## Ethical approval

Patients signed an informed consent and research was ethically approved.

## Consent

Consent done.

## CRediT authorship contribution statement

GG conceived the study and wrote the first draft;

SC revised the manuscript and submitted it

PDC collected figures, elaborated images

DB performed the surgery, collected results, wrote the paper

RN directed the paper, revised the paper, selected and elaborated methods and results

## Declaration of competing interest

None.
